# New statistical model for misreported data with application to current public health challenges

**DOI:** 10.1038/s41598-021-02620-5

**Published:** 2021-12-02

**Authors:** David Moriña, Amanda Fernández-Fontelo, Alejandra Cabaña, Pedro Puig

**Affiliations:** 1 Department of Econometrics, Statistics and Applied Economics, Riskcenter-IREA, Universitat de Barcelona, Barcelona, Spain; 2 Centre de Recerca Matemàtica, Cerdanyola del Vallès, Spain; 3 Chair of Statistics, Humboldt-Universität zu Berlin, Berlin, Germany; 4 Departament de Matemàtiques, Universitat Autònoma de Barcelona, Cerdanyola del Vallès, Spain

**Keywords:** Computational models, Statistical methods, Statistics, Infectious diseases

## Abstract

The main goal of this work is to present a new model able to deal with potentially misreported continuous time series. The proposed model is able to handle the autocorrelation structure in continuous time series data, which might be partially or totally underreported or overreported. Its performance is illustrated through a comprehensive simulation study considering several autocorrelation structures and three real data applications on human papillomavirus incidence in Girona (Catalonia, Spain) and Covid-19 incidence in two regions with very different circumstances: the early days of the epidemic in the Chinese region of Heilongjiang and the most current data from Catalonia.

## Introduction

There has been a growing interest in the past years to deal with data that is only partially registered or underreported in the time series literature. This phenomenon is very common in many fields, and has been previously explored by different approaches in epidemiology, social and biomedical research among many other contexts^1–5^. The sources and underlying mechanisms that cause the underreporting might differ depending on the particular data. Some authors consider a situation where the registry is updated with time and therefore the underreporting issue is mitigated^6^. That leads to temporary underreporting while this work is focused on permanent underreporting, where the registered data are never updated in order to become more accurate. From the methodological point of view, several alternatives have been explored, from Markov chain Monte-Carlo based methods^5^ to recent discrete time series approaches^7,8^. Several attempts to estimate the degree of underreporting in different contexts have been done^9^, although there is a lack of models incorporating continuous time series structures and handling underreporting.

One of the fields where the interest in addressing the underreporting issues is higher is the epidemiology of infectious diseases. In the last few years, many approaches to deal with underreported data have been suggested with a growing level of sophistication from the usage of multiplication factors^10^ to several Markov-based models^11,12^ or even spatio-temporal modelling^13^. Even a new R^14^ package able to fitting endemic-epidemic models based on approximative maximum likelihood to underreported count data has been recently published^15^. This work presents two examples where such phenomenon appears.

Human papillomavirus (HPV) is one of the most prevalent sexually transmitted infections. It is so common that nearly all sexually active people have it at some point in their lives, according to the information provided by the United States’ Centers for Disease Control and Prevention (CDC)^16^. Generally, the infection disappears on its own without inducing any health problem, but in some cases it can produce an abnormal growth of cells on the surface of the cervix that could potentially lead to cervical cancer. HPV infection is also related to other cancers (vulva, vagina, penis, anus, $\ldots$) and other diseases like genital warts (GW). The fact that most cases of HPV infection are asymptomatic causes that public health registries might be potentially underestimating its incidence. The underreporting phenomenon in HPV data from the discrete time series point of view has been recently studied^7^.

There is an enormous global concern around 2019-novel coronavirus (SARS-CoV-2) infection in the last few months, leading the World Health Organization (WHO) to declare public health emergency^17^. As the symptoms of this infection can be easily confused with those of similar diseases like Middle East Respiratory Syndrome Coronavirus (MERS-CoV) or Severe Acute Respiratory Syndrome Coronavirus (SARS-CoV), its incidence has been notably underreported, especially at the beginning of the outbreak in Wuhan (Hubei province, China) by December 2019.

## Methods

The proposed methodology is described in detail in this section, along with an introduction of the real data examples used to illustrate its performance. All the analyses developed to generate the results reported in this paper were conducted in R and the figures were generated using the R packages *ggplot2*^18^ and *ggfortify*^19^.

### Application examples

The first real example, discussed in detail in “Example: HPV infection incidence” section is aimed to analyze the series of weekly cases of HPV infection in Girona in the period 2010–2014. This data set is available from the Health Department of the Catalan Government (https://www.ics.gencat.cat/sisap/diagnosticat/principal?patologia=Papil%B7loma&lang=en). The second example (“Example: Covid-19 incidence in the region of Heilongjiang” section), regarding the daily SARS-CoV-2 infection in the Chinese region of Heilongjiang in the period 2020/01/22–2020/02/26, was collected from the COVID-19 Data Repository by the Center for Systems Science and Engineering (CSSE) at Johns Hopkins University GitHub repository (https://github.com/CSSEGISandData/COVID-19/). The third real example, described in “Example: Covid-19 incidence in Catalonia” section is again focused on Covid-19 infection but in Catalonia in the period 2021/05/16–2021/06/20, and showing a completely different behavior. This data set is freely available from the Health Department of the Catalan Government (https://dadescovid.cat/static/csv/casos_sexe_municipi.zip). These examples were chosen because there is a great consensus among the scientific community that both diseases (HPV and Covid-19) are severely underreported, and the three series present very different behavior, so they allow us to illustrate the performance of the proposed methodology in very different situations.

No data processing was conducted in any case beyond selecting the regions and time periods of interest. The final data sets and R codes used to obtain the described results are available in the Github repository https://github.com/dmorinya/MisRepARMA.

### Model definition

Consider an unobservable process with an AutoRegressive Moving Average (*ARMA*(*p*, *r*)) structure defined by

1 $$\begin{aligned} X_t = \alpha _1 X_{t-1} + \cdots + \alpha _p X_{t-p} + \theta _1 \epsilon _{t-1} + \cdots + \theta _r \epsilon _{t-r} + \epsilon _t, \end{aligned}$$

where $\epsilon _t$ is a Gaussian white noise process with $\epsilon _t \sim N(\mu _{\epsilon }, \sigma _{\epsilon }^2)$. The ARMA processes belong to the family of so called linear processes. Their importance relies on the fact that any stationary nondeterministic process can be written as a sum of a linear process and a deterministic component^20^. These models are very well known, have been used in many applications since their introduction in the early 1950’s and are general and flexible enough to be useful in a wide range of different contexts. Most used statistical software packages include functions that allow straightforward fitting of this family of models, so it seems a natural choice in the present work.

In our setting, this process $X_t$ cannot be directly observed, and all we can see is a part of it, expressed as

2 $$\begin{aligned} Y_t=\left\{ \begin{array}{ll} X_t &{} {\text { with probability }} 1-\omega \\ q \cdot X_t &{} {\text { with probability }} \omega \end{array} \right. \end{aligned}$$

The interpretation of the parameters in Eq. (2) is straightforward: *q* is the overall intensity of misreporting (if $0< q < 1$ the observed process $Y_t$ would be underreported while if $q > 1$ the observed process $Y_t$ would be overreported). The parameter $\omega$ can be interpreted as the overall frequency of misreporting (proportion of misreported observations). The proposed model is a particular case of Hierarchical Mixtures-of-Experts (HME) modelling (see^21,22^ for instance), with an ARMA process instead of a linear model in the hidden layer.

### Model properties

Consider that the unobserved process $X_t$ follows an *ARMA*(*p*, *r*) model as defined in Eq. (1). As can be seen in Appendix 1 (Supplementary Material), the observed process has mean ${\mathbb {E}}(Y_t) = \frac{\mu _{\epsilon }}{1-\alpha _1 - \cdots - \alpha _p} \cdot \left( 1 - \omega + q \cdot \omega \right)$ and variance ${\mathbb {V}}(Y_t) = \left( \left( \frac{\sigma _{\epsilon }^2 \cdot (1+\theta _1^2+ \cdots + \theta _r^2)}{1-\alpha _1^2- \cdots - \alpha _p^2}\right) +\frac{\mu ^2_{\epsilon }}{(1-\alpha _1 - \cdots - \alpha _p)^2}\right) \cdot (1+\omega \cdot (q^2-1)) -\frac{\mu ^2_{\epsilon }}{(1-\alpha _1 - \cdots - \alpha _p)^2} \cdot (1-\omega +q \cdot \omega )^2$. The autocorrelation function of the observed process can be written in terms of the features of the hidden process $X_t$ as

3 $$\begin{aligned} \begin{array}{ll} \rho _{Y}(k) &{} = \frac{V(X_t) (1-\omega +q \omega )^2}{(V(X_t)+E(X_t)^2) (1+\omega (q^2-1))-E(X_t)^2 (1-\omega + q \cdot \omega )^2} \cdot \rho _{X}(k) \\ &{} = c(\alpha _1, \ldots , \alpha _p, \theta _1, \ldots , \theta _r, \mu _{\epsilon }, \sigma ^2_{\epsilon }, \omega , q) \cdot \rho _X(k), \end{array}\end{aligned}$$

where $\rho _X$ is the autocorrelation function of the unobserved process $X_t$.

A situation of particular interest is the case $\omega = 1$, meaning that all the observations might be underreported and that a simpler model for $Y_t$ excluding the parameter $\omega$ might be suitable

4 $$\begin{aligned} Y_t = q \cdot X_t. \end{aligned}$$

In this case, however, the observed process $Y_t$ would be a non-identifiable *ARMA*(*p*, *r*) model as the parameter *q* cannot be estimated on the basis of the methodology described in the following section.

### Estimation

The likelihood function of the observed process $Y_t$ is not easily computable but the parameters of the model can be estimated by means of an iterative algorithm based on its marginal distribution, using the R packages *mixtools*^23^ and *forecast*^24,25^. The main steps are described in detail below:

1. Following Eq. (2), the observed process $Y_t$ can be written as $Y_t = (1-Z_t) \cdot X_t + q \cdot Z_t \cdot X_t$, where $Z_t$ is an indicator of the underreported observations, following a Bernoulli distribution with probability of success $\omega$ $(Z_t \sim Bern(\omega ))$. The marginal distribution of $Y_t$ is a mixture of two normal random variables $N \left( \mu , \sigma ^2 \right)$ and $N \left( q \cdot \mu , q^2 \cdot \sigma ^2 \right)$ respectively, where $\mu = \frac{\mu _{\epsilon }}{1-\alpha _1-\cdots -\alpha _p}$ and $\sigma ^2=\frac{\sigma _{\epsilon }^2 \cdot (1+\theta _1^2 + \cdots + \theta _r^2)}{1-\alpha _1^2- \cdots - \alpha _p^2}$. This fact can be used to obtain initial estimates for *q* and $\omega$. Using the EM algorithm (specifically on the E-step), the posterior probabilities (conditional on the data and the obtained estimates) can be computed. This can be done using, for instance, the R package *mixtools*.
2. Using the indicator $\hat{Z_t}$ obtained in the previous step, the series is divided in two: One including the underreported observations (treating the non-underreported values as missing data) and another with the non underreported observations (treating the underreported values as missing data). An *ARMA* model is fitted to each of these two series and a new $\hat{q}$ is obtained by dividing the fitted means.
3. A mixture of two normals is fitted to the observed series $Y_t$ with mean and standard deviation fixed to the corresponding values obtained from the previous step, and a new $\omega$ is estimated.
4. Steps (ii) and (iii) are repeated until the quadratic distance between two consecutive iterations $(\hat{q}_i-\hat{q}_{i-1})^2+(\hat{\omega }_i-\hat{\omega }_{i-1})^2+\sum _j (\hat{\alpha }_{j_{i}}- \hat{\alpha }_{j_{i-1}})^2 + \sum _k (\hat{\theta }_{k_{i}}- \hat{\theta }_{k_{i-1}})^2$ is below a fixed tolerance level.
5. Once the parameter estimates are stable according to the previous criterion, the underlying process $X_t$ is reconstructed as $\hat{X_t}=(1-\hat{Z_t}) \cdot Y_t + \frac{1}{\hat{q}} \cdot \hat{Z_t} \cdot Y_t$, and an *ARMA* model is fitted to the reconstructed process to obtain $\hat{\alpha }_j$, $j=1, \ldots , p$, $\hat{\theta }_k$, $k=1, \ldots , r$ and $\hat{\sigma _{\epsilon }}^2$.

To account for potential trends or seasonal behaviour, covariates can be included in the described estimation process expressing the observed series as $Y_t = \beta _0 + \beta _1 C_1 + \cdots \beta _k C_k + (1-Z_t) \cdot X_t + q \cdot Z_t \cdot X_t$, where $C_1, \ldots , C_k$ are the covariates, so its stationarity is ensured. The $\beta _i$ coefficients, $i=1, \ldots , k$, can be estimated by Ordinary Least Squares (OLS). Additionally, a parametric bootstrap procedure with 500 replicates is used to estimate standard errors and build confidence intervals based on the percentiles of the distribution of the estimates. In order to make the described methodology easily accessible to statisticians and data scientists, it has been compiled in the form of the R package *MisRepARMA*^26^. Additionally, non expert users facing this issue can also use an adapted version of the package through the web application https://dmorina.shinyapps.io/MisRepARMA/.

## Results

The results of the proposed methodology over a comprehensive simulation study and an application on two real data sets are shown in this Section.

### Simulation study

A thorough simulation study has been conducted to ensure that the model behaves as expected, including *AR*(*p*), *MA*(*r*) and *ARMA*(*p*, *r*) for $1 \le p, r \le 3$ structures for the hidden process $X_t$ with values for the parameters $\alpha$, $\theta$, *q* and $\omega$ ranging from 0.1 to 0.9 for each parameter (some combinations of parameters have been omitted for $p>1$ or $r>1$ to ensure stationarity). For *ARMA*(*p*, *r*) structures with $p>1$ or $r>1$ the parameters covered the same range (0.1 to 0.9) but with a difference of 0.2 instead of 0.1 for computational feasibility. Only average absolute bias, interval coverage and 95% confidence interval corresponding to $p=r=1$ are shown in Table 1, as higher order models behave in a very similar manner (see Supplementary Material for details). These values are averaged over all combinations of parameters. Additionally, standard *AR*(1), *MA*(1) and *ARMA*(1, 1) models were fitted to the same simulated series without accounting for their underreporting structure.

**Table 1:** Model performance measures (average absolute bias, average interval length and average coverage) summary based on a simulation study.

Structure	Parameter	Bias	AIL	Coverage (%)
*AR*(1)	$\hat{\alpha }$	0.004	0.100	94.92
	$\hat{q}$	$<10^{-3}$	$<10^{-3}$	93.14
	$\hat{\omega }$	$<10^{-3}$	0.050	93.69
Standard *AR*(1)	$\hat{\alpha }$	0.500	0.124	0.96
*MA*(1)	$\hat{\theta }$	$<10^{-3}$	0.116	96.02
	$\hat{q}$	$<10^{-3}$	$<10^{-3}$	94.79
	$\hat{\omega }$	− 0.001	0.050	90.26
Standard *MA*(1)	$\hat{\theta }$	0.499	0.124	1.23
*ARMA*(1, 1)	$\hat{\alpha }$	0.003	0.161	95.66
	$\hat{\theta }$	0.005	0.211	96.97
	$\hat{q}$	$<10^{-3}$	0.001	94.91
	$\hat{\omega }$	$<10^{-3}$	0.050	94.06
Standard *ARMA*(1, 1)	$\hat{\alpha }$	0.492	3.056	52.48
	$\hat{\theta }$	0.509	3.055	51.14

For each autocorrelation structure and parameters combination, a random sample of size $n = 1000$ has been generated using the function *arima.sim* from R package *forecast*^24,25^. Different sample sizes ($n = 50, 100, 500$) have also been considered to study the impact of sample size on accuracy and the results are reported in the Supplementary Material. The performance of the proposed methodology is summarised in Tables S1–S4 for $n = 50, 100, 500$ and 1000 respectively. Average absolute bias is similar regardless of the sample size, while average interval lengths (AIL) are higher and interval coverages are poorer (around 75% for $n = 50$) for lower sample sizes as could be expected. Several bootstrap sizes ($b = 20, 50, 100, 500$) were also considered and the difference between them were negligible, so only results corresponding to $b = 500$ bootstrap replicates are reported.

It is clear from Table 1 that ignoring the underreported nature of data (labeled as *Standard* models in the table) leads to highly biased estimates with extremely low coverage rates, even with larger average interval lengths. This is especially relevant when the intensity or frequency of underreported observations is high.

### Example: HPV infection incidence

**Figure 1:**
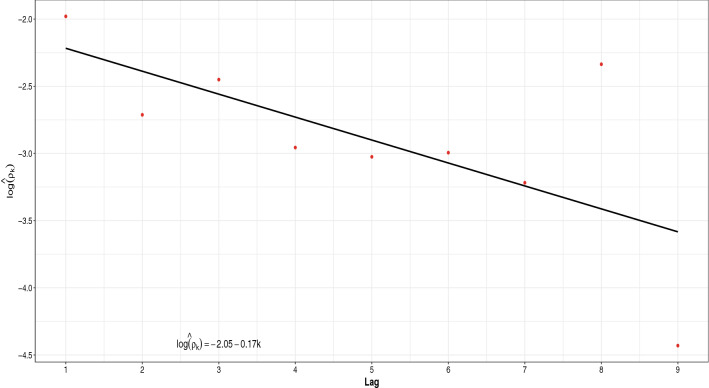
Sample autocorrelation coefficients (red points) and estimated regression line (black solid line) of $\log (\rho _Y(k)) = \log (c)+k \cdot \log (\alpha )$.

The series of weekly cases of HPV infection in Girona in the period 2010–2014 was previously analyzed as a discrete *INAR*(1) hidden Markov process^7^. In a similar way, we aim to analyze the corresponding series of incidence, and an AR process of order 1 seems to be adequate (see Fig. 1). Additionally, the *AR*(1) structure has the lowest AIC when compared to similar alternative models like *AR*(2), *ARMA*(1, 1) and *MA*(1) (AICs are 299.31, 300.47, 300.49 and 299.68 respectively). According to Eq. (3), the autocorrelation function of the observed process $Y_t$ when the hidden process $X_t$ has an *AR*(1) structure takes the form $\rho _Y(k) = c \cdot \alpha ^k$, where

$c = c(\alpha , \mu _{\epsilon }, \sigma ^2_{\epsilon }, \omega , q) = \frac{(1-\omega +q \cdot \omega )^2 \cdot \sigma ^2_{\epsilon }}{(1-\alpha ^2) \cdot \left( \left( \frac{\sigma ^2_{\epsilon }}{1-\alpha ^2}+\frac{\mu ^2_{\epsilon }}{(1-\alpha ^2)}\right) \cdot (1+\omega \cdot (q^2-1)) - (1-\omega +q \cdot \omega )^2 \cdot \frac{\mu _{\epsilon }^2}{(1-\alpha )^2} \right) }$. In particular, in this case we can write $\log (\rho _Y(k)) = \log (c)+k \cdot \log (\alpha )$, so a statistically significant intercept of this linear regression model (estimating the parameters by ordinary least squares method) could be interpreted as an evidence of underreporting, as in this case ($p-value = 0.0014$). It is clear from Fig. 1 that the estimated regression line does not cross the origin, so the behavior of the observed process is consistent with an underlying underreported *AR*(1) process.

By means of the estimation method described in “Estimation” section, it can be seen that the estimated model for the hidden process is $X_t = 0.109 \cdot X_{t-1} + \epsilon _t$, being the observed process $Y_t$,

5 $$\begin{aligned} Y_t = {\left\{ \begin{array}{ll} X_t &{} {\text {with probability }} 0.17 \\ 0.238 \cdot X_t &{} {\text {with probability }} 0.83 \end{array}\right. } \end{aligned}$$

The estimated parameters are reported in Table 2.

**Table 2:** Bootstrap means and standard errors of the proposed model for the HPV example.

Parameter	Bootstrap mean	Bootstrap SE
$\hat{\mu _{\epsilon }}$	0.575	0.100
$\hat{\alpha }$	0.114	0.056
$\hat{\omega }$	0.832	0.135
$\hat{q}$	0.238	0.068

These results are highly consistent with those previously reported in the literature for the number of HPV cases obtained through a discrete time series approach^7^ and can be interpreted in a very straightforward way. Moreover, this new methodology can be used to model the incidence of the disease instead of the number of cases, accounting for potential changes in the underlying population.

**Figure 2:**
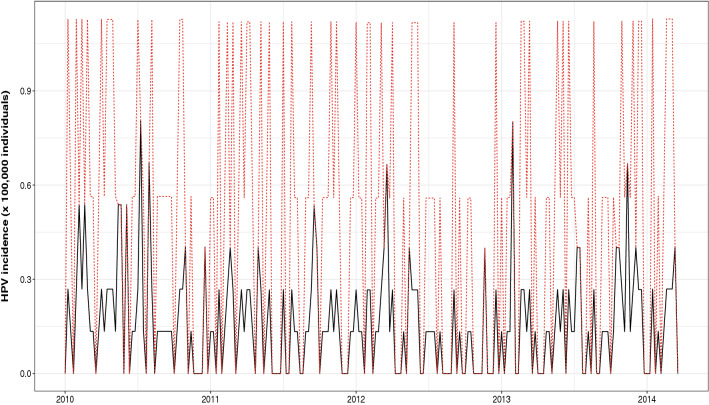
Registered (black solid line) and estimated (red dotted line) HPV incidence in Girona in the period 2010–2014.

The estimated intensity of underreporting is $\hat{q} = 0.238$, with 95% confidence interval (0.106, 0.371). The registered and estimated evolution of HPV incidence within the study period (2010–2014) can be seen in Fig. 2.

These results indicate that only 33% of the estimated HPV incidence in the considered period of time was actually recorded. Taking into account that public health cervical cancer prevention strategies are often designed on the basis of simulation models which are calibrated to registered HPV data^27^, it is clear that providing decision makers with accurate data on HPV incidence is key to ensure optimal allocation of scarce public health funds.

### Example: Covid-19 incidence in the region of Heilongjiang

**Figure 3:**
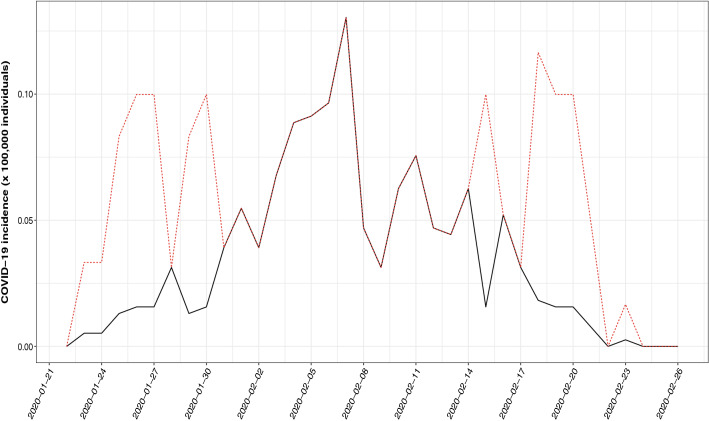
Registered (black solid line) and estimated (red dotted line) COVID-19 incidence in the region of Heilongjiang in the period 2020/01/22–2020/02/26.

The betacoronavirus SARS-CoV-2 has been identified as the causative agent of an unprecedented world-wide outbreak of pneumonia starting in December 2019 in the city of Wuhan (China)^17^, named as Covid-19. Considering that many cases run without developing symptoms beyond those of MERS-CoV, SARS-CoV or pneumonia due to other causes, it is reasonable to assume that the incidence of this disease has been underregistered, especially at the beginning of the outbreak^28^. This section focuses on the Covid-19 incidence registered in Heilongjiang province (north-eastern China) in the period (2020/01/22–2020/02/26), and it can be seen in Fig. 3 that the registered data (black color) reflect only a fraction of the estimated actual incidence (red color).

**Figure 4:**
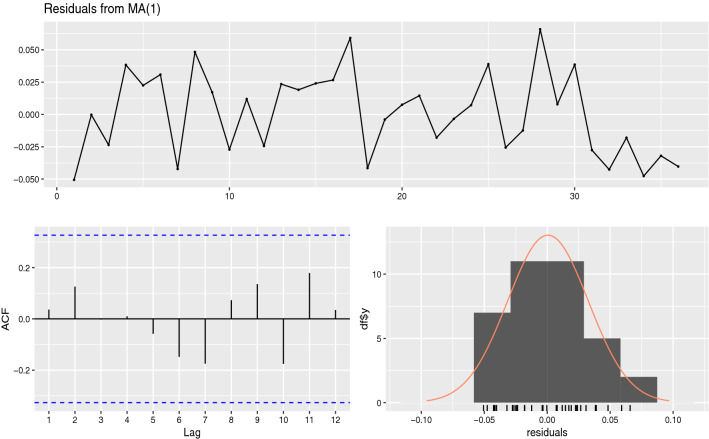
Residual analysis (raw residuals (upper graph), autocorrelation coefficients (lower graph left) and histogram (lower graph right)) after fitting a *MA*(1) model to the Heilongjiang COVID-19 data.

Another respiratory disease caused by a coronavirus (MERS-CoV) has been modeled in a previous work as an *ARMA*(3, 1)^29^, so we evaluated the performance of this model and similar ones. Probably due to the shortness of the available data this autoregressive structure was not observed and in our case the best performing model was an *MA*(1) (AIC of -140.17 against -136.1 for the *ARMA*(3, 1)), consistently with the residuals profile shown in Fig. 4, obtained from fitting an *MA*(1) model to the most likely process $X_t$ reconstructed following step (v) in “Estimation” section.

By means of the estimation method described in “Estimation” section, it can be seen that the estimated model for the hidden process is $X_t = 0.528 \cdot \epsilon _{t-1} + \epsilon _t$, being the observed process $Y_t$,

6 $$\begin{aligned} Y_t = {\left\{ \begin{array}{ll} X_t &{} {\text {with probability }} 0.564 \\ 0.157 \cdot X_t &{} {\text {with probability }} 0.436 \end{array}\right. } \end{aligned}$$

The estimated parameters are reported in Table 3.

**Table 3:** Bootstrap means and standard errors of the proposed model for the Heilongjiang Covid-19 example.

Parameter	Bootstrap mean	Bootstrap SE
$\hat{\mu _{\epsilon }}$	0.057	0.012
$\hat{\theta }$	0.528	0.173
$\hat{\omega }$	0.436	0.160
$\hat{q}$	0.157	0.076

### Example: Covid-19 incidence in Catalonia

**Figure 5:**
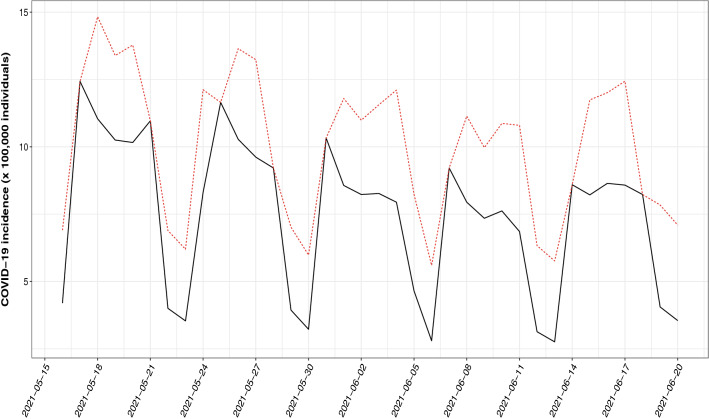
Registered (black solid line) and estimated (red dotted line) COVID-19 incidence in Catalonia in the period 2021/05/16–2021/06/20.

The Covid-19 incidence in Catalonia in the period 2021/05/16–2021/06/20 looks totally different. As it can be seen in Fig. 5, these data present a slight decreasing trend and weekly seasonality. The decreasing trend is probably a consequence of a successful vaccination campaign, while the weekly seasonality is artificially created by issues in the notification process, as it can be seen that the lower number of cases are consistently observed by the weekends, while the peak each week is observed on Mondays. In order to account for the trend the simple linear regression model $f(t) = \beta _0 + \beta _1 \cdot t$ was included as a covariate and the following trigonometric function was used to incorporate the observed periodic behaviour.

7 $$\begin{aligned} g(t) = \beta _2 \cdot \sin \left( \frac{2 \cdot \pi \cdot t}{7}\right) + \beta _3 \cdot \cos \left( \frac{2 \cdot \pi \cdot t}{7}\right) \end{aligned}$$

In this case, the best fitting model according to AIC and residuals profile is an AR(2).

As shown in Table 4, the estimates related to underreporting reveal a lower intensity (although a higher frequency probably related to its periodicity) of the issue compared to the previous example, as could be expected. In fact, $\hat{\alpha _1}$ is not significantly different to zero, so a simpler model for uncorrelated misreported data like the one proposed in^30^ might be enough.

**Table 4:** Bootstrap means and standard errors of the proposed model for the Catalonia Covid-19 example.

Parameter	Bootstrap mean	Bootstrap SE
$\hat{\beta _0}$	11.513	1.251
$\hat{\beta _1}$	− 0.078	0.013
$\hat{\beta _2}$	− 1.037	0.246
$\hat{\beta _3}$	− 2.599	0.234
$\hat{\alpha _1}$	0.0173	0.184
$\hat{\alpha _2}$	− 0.372	0.187
$\hat{\omega }$	0.782	0.230
$\hat{q}$	0.712	0.089

## Discussion

In biomedical and epidemiological research, the usage of disease registries in order to analyze the impact and incidence of health issues is very common. However, the accuracy and data quality of such registries is in many cases at least doubtful. This is the case, for instance, for rare diseases^31^ or health issues that clear asymptomatically in most cases like HPV infection. In the case of HPV incidence in Girona in the period 2010–2014, the registered weekly average is 0.17 cases per 100,000 individuals, while the reconstructed process has a weekly average of 0.51 cases per 100,000 individuals, showing that only 33% of the estimated real incidence is recorded by the public health system. It must be considered that HPV infection is related to subsequent complications such as cervical cancer in some cases and that public health cervical cancer prevention strategies are often designed on the basis of simulation models which are calibrated to registered HPV data^27^ and therefore the optimal allocation of scarce public health resources cannot be ensured if the under-reporting issue is not accounted for. This result is very consistent with that of^7^, where the authors claim that only 38% of the HPV cases were registered in the same area and period of time.

The Heilongjiang region Covid-19 data reveal that in average about 60% of the estimated actual incidence in the period 2020/01/22–2020/02/26 was reported. The unavailable data estimated by the proposed methodology are crucial to provide public health decision-makers with reliable information, which can also be used to improve the accuracy of dynamic models aimed to estimate the spread of the disease^28^. In China and almost globally afterwards, different non-pharmaceutical interventions were undertaken in order to minimise the impact of the disease on the general population and especially over the health systems, which were put to the limit of their capacity by the pandemic. In this context, one of the main challenges in predicting the evolution of the disease or evaluating the impact of these strategies is to use data as accurate as possible, taking into account that many Covid-19 cases are asymptomatic or with mild symptoms and a generalized shortage of testing kits^32^, and therefore knowing that the registered number of affected individuals might be severely underestimated. The analysis of Covid-19 incidence in a completely different context (very recent daily data from a European region) shows that the model behaves as expected and is capable of handling trends and seasonality. In the Catalan case, the model reveals that more than 74% of the cases in the period 2021/05/16–2021/06/20 were registered. These examples are only used to illustrate the performance of the proposed methodology, but to properly analyze the evolution of an infectious disease with the behaviour shown by Covid-19 models that take the spreading dynamics into account are probably more appropriate (see^33,34^ for instance).

The concerns around accuracy of registered data have recently led to the publication of recommendations to improve data collection to ensure accuracy of registries (see for instance^35,36^). Nonetheless, these recommendations are very recent and may be difficult for the public health services of many countries to fully implement them, due to operational or structural issues.

The proposed methodology is able to deal with underreported (or overreported) data in a very natural and straightforward way, estimating its intensity and frequency on a continuous time series, and allowing to reconstruct the most likely unobserved process. It is also flexible enough to handle covariates straightforwardly, and therefore it is simple to introduce trends or seasonality if necessary, so it can be useful in many contexts, where these issues might arise.

The simulation study shows that the proposed methodology behaves as expected and that the parameters used in the simulations, under different autocorrelation structures, are properly recovered, regardless of the intensity and frequency of the underreporting issues. It also reveals that using standard time series models can lead to severely biased estimates and low coverage rates, while the proposed methodology can overcome the issue of underreporting and provide unbiased and efficient inference.

The methods introduced in this paper could certainly be considered as a starting point to develop more general methods, able to deal with non-stationary continuous time series, adapting the ideas developed in^33^ for the discrete case. From the applied point of view, it would be very interesting to use these kind of models to analyze other issues that might be potentially underreported and to analyze more thoroughly the examples used to illustrate the performance of the discussed models.

## Supplementary Information

Supplementary Information 1.

Supplementary Information 2.

## Data Availability

All data generated or analyzed during this study are included in this published article (and its Supplementary Information files).
