# Supplementary material for: New statistical model for misreported data with application to current public health challenges
Source: Sci Rep. 2021 Dec 2;11:23321. doi: 10.1038/s41598-021-02620-5 (PMC8640038; doi:10.1038/s41598-021-02620-5)
Supplement: Supplementary file 1 — Supplementary Information 1. [file 41598_2021_2620_MOESM1_ESM.pdf]

Package ‘MisRepARMA’

July 14, 2021

Type Package

Title Misreported Time Series Analysis

Version 0.0.2

Date 2021-07-14

Encoding UTF-8

Maintainer David Moriña Soler <dmorina@ub.edu>

Description Provides a simple and trustworthy methodology for the analysis of misreported continuous time series. See Moriña, D, Fernández-Fontelo, A, Cabaña, A, Puig P. (2021) <arXiv:2003.09202v2>.

Depends R (>= 3.5.0), mixtools, boot, tseries

License GPL (>= 2)

NeedsCompilation no

Author David Moriña Soler [aut, cre] (<https://orcid.org/0000-0001-5949-7443>), Amanda Fernández-Fontelo [aut], Alejandra Cabaña [aut], Pedro Puig [aut]

R topics documented:

MisRepARMA-package 1
fitMisRepARMA 2
reconstruct 4

Index **5**

MisRepARMA-package *Misreported time series analysis*

Description

Provides a simple and trustworthy methodology for the analysis of misreported continuous time series. See Moriña, D, Fernández-Fontelo, A, Cabaña, A, Puig P. (2021) <https://arxiv.org/abs/2003.09202v2>.

Details

Package:	MisRepARMA
Type:	Package
Version:	0.0.2
Date:	2021-07-14
License:	GPL version 2 or newer
LazyLoad:	yes

The package implements function `fitMisRepARMA`, which is able to fit an ARMA time series model to misreported data, and the function `reconstruct` which is able to reconstruct the most likely real series.

Author(s)

David Moriña, Amanda Fernández-Fontelo, Alejandra Cabaña, Pedro Puig

Mantainer: David Moriña Soler <dmorina@ub.edu>

References

Davison, A.C. and Hinkley, D.V. (1997) Bootstrap Methods and Their Application. Cambridge University Press.

Kunsch, H.R. (1989) The jackknife and the bootstrap for general stationary observations. Annals of Statistics, **17**, 1217–1241.

Moriña, D., Fernández-Fontelo, A., Cabaña, A., Puig, P. (2021): New statistical model for misreported data with application to current public health challenges. arXiv preprint (https://arxiv.org/pdf/2003.09202.pdf)

Politis, D.N. and Romano, J.P. (1994) The stationary bootstrap. Journal of the American Statistical Association, **89**, 1303–1313.

See Also

`MisRepARMA-package`, `fitMisRepARMA`, `reconstruct`

`fitMisRepARMA` *Fit ARMA model to misreported time series data*

Description

Fits an ARMA model to misreported time series data.

Usage

```
fitMisRepARMA(y, tol, B, p_AR, q_MA, covars=NULL, misReport="U", ...)
```

Arguments

`y`	a numeric vector or time series giving the original data.
`tol`	tolerance limit to stop the iterative algorithm.
`B`	the number of bootstrap series to compute.
`p_AR`	order of the AR part.
`q_MA`	order of the MA part.
`covars`	matrix of explanatory variables. Its default value is `NULL`.
`misReport`	direction of misreporting issue. Its default value is `U` for underreported data, can also take the value `O` for overreported data.
`...`	additional arguments to pass to `tsboot`, for instance those regarding parallelization.

Details

The model based resampling scheme with `B` bootstrap resamples is computed. This

Value

An object of class `fitMisRepARMA` with the following elements is returned:

- `data`: The original time series.
- `t0`: The results of applying statistic to the original series.
- `t`: Estimates on each replicated time series.
- `call`: The original call to tsboot.

Author(s)

David Moriña, Amanda Fernández-Fontelo, Alejandra Cabaña, Pedro Puig

References

Davison, A.C. and Hinkley, D.V. (1997) Bootstrap Methods and Their Application. Cambridge University Press.

Kunsch, H.R. (1989) The jackknife and the bootstrap for general stationary observations. Annals of Statistics, **17**, 1217–1241.

Moriña, D., Fernández-Fontelo, A., Cabaña, A., Puig, P. (2021): New statistical model for misreported data with application to current public health challenges. arXiv preprint (https://arxiv.org/pdf/2003.09202.pdf)

Politis, D.N. and Romano, J.P. (1994) The stationary bootstrap. Journal of the American Statistical Association, **89**, 1303–1313.

See Also

`MisRepARMA-package`, `reconstruct`

Examples

```
### Simulate underreported time series data
set.seed(12345)
x <- arima.sim(model=list(ar=0.4), n=50)
ind <- rbinom(50, 1, 0.6)
y <- ifelse(ind==0, x, x*0.3)
mod <- fitMisRepARMA(y, 1e-6, 3, 0.05, 1, 0, covars=NULL, misReport="U")
```

`reconstruct` *Reconstruct the most likely series*

Description

Reconstructs the most likely series.

Usage

```
reconstruct(object)
```

Arguments

`object`	object of class `fitMisRepARMA`.

Value

the function returns a vector of the same length of `data` containing the reconstruction of the most likely series.

Author(s)

David Moriña, Amanda Fernández-Fontelo, Alejandra Cabaña, Pedro Puig

References

D. Moriña, A. Fernández-Fontelo, A. Cabaña, P. Puig (2021): New statistical model for misreported data with application to current public health challenges. arXiv preprint (https://arxiv.org/pdf/2003.09202.pdf)

Davison, A. C. and Hinkley, D. V. (1997) Bootstrap Methods and Their Applications. Cambridge University Press, Cambridge. ISBN 0-521-57391-2

See Also

`MisRepARMA-package`, `fitMisRepARMA`

Examples

```
### Simulate underreported time series data
x <- arima.sim(model=list(ar=0.4), n=50)
ind <- rbinom(50, 1, 0.6)
y <- ifelse(ind==0, x, x*0.3)
pr <- fitMisRepARMA(y, 1e-8, 5, 0.05, 1, 0, covars=NULL, misReport="U")
x <- reconstruct(pr)
```

Index

∗ **MisRepARMA**
fitMisRepARMA, 2
MisRepARMA-package, 1
reconstruct, 4

fitMisRepARMA, *2*, 2, *4*

MisRepARMA *(*MisRepARMA-package*)*, 1
MisRepARMA-package, 1

reconstruct, *2, 3*, 4
