# Supplementary material for: New statistical model for misreported data with application to current public health challenges
Source: Sci Rep. 2021 Dec 2;11:23321. doi: 10.1038/s41598-021-02620-5 (PMC8640038; doi:10.1038/s41598-021-02620-5)
Supplement: Supplementary file 2 — Supplementary Information 2. [file 41598_2021_2620_MOESM2_ESM.pdf]

SUPPLEMENTARY MATERIAL

Accompanying the manuscript:

New statistical model for misreported data with application to current public health challenges

David Moriña
Dept. Econometrics, Statistics and Applied Economics, Riskcenter-IREA, Universitat de Barcelona
Centre de Recerca Matemàtica (CRM)
E-mail: dmorina@ub.edu

Amanda Fernández-Fontelo
Chair of Statistics, School of Business and Economics, Humboldt-Universität zu Berlin, Berlin, Germany

Alejandra Cabaña
Departament de Matemàtiques, Universitat Autònoma de Barcelona

Pedro Puig
Departament de Matemàtiques, Universitat Autònoma de Barcelona
Centre de Recerca Matemàtica (CRM)

1. Appendix 1.

The observed process Y can be expressed as $Y_t = (1-Z_t)\cdot X_t + Z_t \cdot q \cdot X_t$, where $Z_t \sim Bern(\omega)$, and therefore, taking into account that $E(X_t) = \frac{\mu_\epsilon}{1-\alpha_1-\cdots-\alpha_p}$ and $V(X_t) = \frac{\sigma_\epsilon^2\cdot(1+\theta_1^2+\cdots+\theta_r^2)}{1-\alpha_1^2-\cdots-\alpha_p^2}$ and that X_t and Z_t are independent,

$E(Y_t) = E(X_t)\cdot E\big(Z_t + q\cdot(1-Z_t)\big) = \frac{\mu_\epsilon}{1-\alpha_1-\cdots-\alpha_p}\cdot(1-\omega+q\cdot\omega)$.

To compute the variance of the observed process Y_t it is important to observe that $E(Y_t^2) = E(X_t^2)\cdot E((1-Z_t+qZ_t)^2)$. As $E(X_t^2) = \left(\frac{\mu_\epsilon}{1-\alpha_1-\cdots-\alpha_p}\right)^2 + \frac{\sigma_\epsilon^2\cdot(1+\theta_1^2+\cdots+\theta_r^2)}{1-\alpha_1^2-\cdots-\alpha_p^2}$, we only need to compute $E((1-Z_t+qZ_t)^2)$:

$(1-Z_t+qZ_t)^2 = 1 + Z_t^2 - 2Z_t + q^2Z_t^2 = 1 + Z_t(q^2-1)$,

and so $E((1-Z_t+qZ_t)^2) = 1+\omega\cdot(q^2-1)$. From here, $E(Y_t^2) = \left(\left(\frac{\mu_\epsilon}{1-\alpha_1-\cdots-\alpha_p}\right)^2 + \frac{+\sigma_\epsilon^2\cdot(1+\theta_1^2+\cdots+\theta_r^2)}{1-\alpha_1^2-\cdots-\alpha_p^2}\right)\cdot\big(1+\omega\cdot(q^2-1)\big)$ and $\big(E(Y_t)\big)^2 = \left(\frac{\mu_\epsilon}{1-\alpha_1-\cdots-\alpha_p}\right)^2\cdot(1-\omega+q\omega)^2$.

Therefore,

$$V(Y_t) = E(Y_t^2) - \big(E(Y_t)\big)^2$$
$$= \left(\left(\frac{\sigma_\epsilon^2\cdot(1+\theta_1^2+\cdots+\theta_r^2)}{1-\alpha_1^2-\cdots-\alpha_p^2}\right) + \frac{\mu_\epsilon^2}{\big(1-\alpha_1-\cdots-\alpha_p\big)^2}\right)\cdot\big(1+\omega\cdot(q^2-1)\big)$$
$$- \frac{-\mu_\epsilon^2}{\big(1-\alpha_1-\cdots-\alpha_p\big)^2}\cdot(1-\omega+q\cdot\omega)^2.$$

The covariance of the hidden process X_t can be written as[1]

$\gamma_X(k) = \sigma_\epsilon^2\sum_{j=0}^{\infty}\psi_j\psi_{j+|k|}$,

where $\psi(z) = \sum_{j=0}^{\infty}\psi_j z^j = \frac{\theta(z)}{\alpha(z)}$ for $|z|\le 1$, with $\theta(z) = 1+\theta_1\cdot z+\cdots+\theta_r\cdot z^r$ and $\alpha(z) = 1+\alpha_1\cdot z+\cdots+\alpha_p\cdot z^p$. Considering $Z_t \sim Bern(\omega)$ we can write

$$E(Y_tY_{t+k}) = E\big(X_t\cdot(1-Z_t)X_{t+k}\cdot(1-Z_{t+k})\big) + E(X_t\cdot(1-Z_t)qX_{t+k}\cdot Z_{t+k})$$
$$+ E(X_{t+k}\cdot(1-Z_{t+k})qX_t\cdot Z_t) + E(qX_tqX_{t+k}\cdot Z_tZ_{t+k})$$

As Z_t and Z_{t+k} are independent of \{X_j\} and both have expectation ω,

$E\big(X_t\cdot(1-Z_t)X_{t+k}(1-Z_{t+k})\big) = (1-\omega)^2E(X_tX_{t+k})$,
$E(X_t\cdot(1-Z_t)qX_{t+k}\cdot Z_{t+k}) = (1-\omega)\omega qE(X_tX_{t+k})$,
$E(X_{t+k}\cdot(1-Z_{t+k})qX_t\cdot Z_t) = (1-\omega)q\omega E(X_tX_{t+k})$,
$E(qX_tqX_{t+k}\cdot Z_tZ_{t+k}) = \omega^2q^2E(X_tX_{t+k})$.

Therefore, $E(Y_tY_{t+k}) = (1-\omega+q\cdot\omega)^2E(X_tX_{t+k})$. From here, the covariance of the observed process Y_t is

$$\gamma_Y(k) = Cov(Y_t, Y_{t+k}) = (1-\omega+q\cdot\omega)^2\cdot$$
$$\left(\gamma_X(k) + \frac{\mu_\epsilon^2}{\big(1-\alpha_1-\cdots-\alpha_p\big)^2}\right) - \left(\frac{\mu_\epsilon}{1-\alpha_1-\cdots-\alpha_p}\right)^2\cdot(1-\omega+q\cdot\omega)^2 = (1-\omega+q\cdot\omega)^2\cdot\gamma_X(k).$$

Finally, the autocorrelation of Y_t can be expressed as

$$\rho_Y(k) = \frac{\gamma_Y(k)}{V(Y_t)} = \frac{\gamma_X(k)\cdot(1-\omega+q\cdot\omega)^2}{(V(X_t)+E(X_t)^2)\cdot\left(1+\omega\cdot(q^2-1)\right)-E(X_t)^2\cdot(1-\omega+q\cdot\omega)^2} = \frac{V(X_t)\cdot\rho_X(k)\cdot(1-\omega+q\cdot\omega)^2}{(V(X_t)+E(X_t)^2)\cdot\left(1+\omega\cdot(q^2-1)\right)-E(X_t)^2\cdot(1-\omega+q\cdot\omega)^2},$$

where $\rho_X(k)$ is the autocorrelation function of the hidden process X_t.

2. Supplementary tables

Structure	Parameter	Bias	AIL	Coverage (%)
AR(1)	α	0.068	0.469	64.75%
	q	-0.002	0.015	68.04%
	ω	-0.002	0.213	65.43%
MA(1)	θ	0.026	0.601	70.78%
	q	< 0.0001	0.019	69.82%
	ω	-0.002	0.208	66.26%
ARMA(1, 1)	α	0.082	0.876	71.85%
	θ	0.051	0.990	69.44%
	q	-0.001	0.027	71.06%
	ω	-0.001	0.221	65.74%
AR(2)	α_1	0.037	0.504	66.80%
	α_2	0.08	0.506	65.77%
	q	< 0.0001	0.014	71.15%
	ω	< 0.0001	0.206	65.56%
AR(3)	α_1	0.044	0.513	88.16%
	α_2	0.068	0.514	88.22%
	α_3	0.054	0.517	89.81%
	q	-0.001	0.012	94.01%
	ω	< 0.0001	0.204	86.72%
MA(2)	θ_1	0.014	0.603	70.38%
	θ_2	0.007	0.744	72.12%
	q	-0.001	0.016	69.14%
	ω	-0.002	0.207	65.27%
MA(3)	θ_1	0.042	0.623	90.64%
	θ_2	0.009	0.806	94.65%
	θ_3	0.043	0.835	94.77%
	q	-0.001	0.013	92.78%
	ω	< 0.0001	0.204	86.69%
ARMA(2, 1)	α_1	-0.037	1.953	64.30%
	α_2	0.121	1.226	59.69%
	θ	0.113	2.132	64.05%
	q	-0.001	0.020	64.30%
	ω	< 0.0001	0.214	56.62%
ARMA(2, 2)	α_1	-0.018	2.117	97.98%
	α_2	0.149	1.572	93.69%
	θ_1	0.103	2.351	97.61%
	θ_2	0.002	1.284	94.89%
	q	-0.002	0.030	96.96%
	ω	0.001	0.221	87.63%

ARMA(3, 1)	α_1	0.055	1.870	99.12%
	α_2	0.065	1.069	93.86%
	α_3	0.044	0.680	92.73%
	θ	0.033	2.068	97.93%
	q	-0.002	0.021	95.86%
	ω	-0.001	0.213	85.71%
ARMA(3, 2)	α_1	0.057	2.527	98.25%
	α_2	0.135	1.989	96.89%
	α_3	-0.001	1.475	96.17%
	θ_1	0.020	2.862	98.11%
	θ_2	-0.073	2.287	98.78%
	q	-0.006	0.032	96.81%
	ω	-0.001	0.221	86.69%
ARMA(1, 2)	α_1	0.099	1.270	97.76%
	θ_1	0.046	1.418	94.64%
	θ_2	0.028	1.011	93.81%
	q	-0.002	0.035	95.86%
	ω	< 0.0001	0.237	87.96%
ARMA(1, 3)	α_1	0.095	1.476	97.12%
	θ_1	0.095	1.652	93.90%
	θ_2	0.029	1.220	93.32%
	θ_3	0.073	1.047	90.86%
	q	-0.003	0.073	96.05%
	ω	< 0.0001	0.244	87.26%
ARMA(2, 3)	α_1	-0.058	2.341	97.72%
	α_2	0.181	1.727	94.13%
	θ_1	0.180	2.602	96.77%
	θ_2	-0.008	1.544	96.69%
	θ_3	0.091	1.202	90.47%
	q	-0.002	0.045	97.32%
	ω	-0.001	0.228	88.18%
ARMA(3, 3)	α_1	0.030	2.315	98.38%
	α_2	0.131	1.850	97.47%
	α_3	0.024	1.718	98.63%
	θ_1	0.086	2.639	98.06%
	θ_2	-0.027	2.308	98.89%
	θ_3	0.083	1.452	94.16%
	q	-0.003	0.045	97.25%
	ω	-0.001	0.229	87.96%

Table S1. Model performance measures summary based on a simulation study (simulated sample sizes of *n = 50* observations).

Structure	Parameter	Bias	AIL	Coverage (%)
AR(1)	α	0.039	0.334	84.36%
	q	< 0.0001	0.008	86.56%
	ω	< 0.0001	0.153	84.22%

MA(1)	θ	0.010	0.392	87.24%
	q	< 0.0001	0.009	85.19%
	ω	< 0.0001	0.152	83.13%
ARMA(1, 1)	α	0.037	0.581	87.38%
	θ	0.047	0.693	85.29%
	q	< 0.0001	0.014	86.53%
	ω	< 0.0001	0.162	83.30%
AR(2)	α_1	0.017	0.350	83.47%
	α_2	0.039	0.362	83.23%
	q	< 0.0001	0.005	85.63%
	ω	< 0.0001	0.150	82.10%
AR(3)	α_1	0.018	0.370	90.55%
	α_2	0.031	0.365	90.64%
	α_3	0.025	0.372	92.49%
	q	< 0.0001	0.005	93.64%
	ω	< 0.0001	0.149	89.22%
MA(2)	θ_1	0.007	0.379	86.95%
	θ_2	0.004	0.450	88.25%
	q	< 0.0001	0.008	84.19%
	ω	< 0.0001	0.152	82.81%
MA(3)	θ_1	0.030	0.391	87.81%
	θ_2	0.006	0.473	92.62%
	θ_3	0.032	0.487	92.24%
	q	< 0.0001	0.006	92.65%
	ω	< 0.0001	0.151	89.75%
ARMA(2, 1)	α_1	-0.050	1.657	83.76%
	α_2	0.083	1.072	80.16%
	θ	0.089	1.733	83.68%
	q	< 0.0001	0.012	81.84%
	ω	< 0.0001	0.157	78.56%
ARMA(2, 2)	α_1	-0.011	1.601	98.65%
	α_2	0.064	1.243	97.10%
	θ_1	0.064	1.684	98.21%
	θ_2	0.038	0.803	94.07%
	q	< 0.0001	0.023	97.14%
	ω	0.001	0.166	91.44%
ARMA(3, 1)	α_1	0.037	1.547	98.85%
	α_2	0.024	0.986	96.64%
	α_3	0.017	0.501	93.87%
	θ	0.014	1.630	97.74%
	q	< 0.0001	0.018	96.13%
	ω	0.002	0.160	91.52%
ARMA(3, 2)	α_1	0.059	2.232	98.88%
	α_2	0.057	1.764	98.06%
	α_3	-0.021	1.358	98.21%
	θ_1	-0.010	2.368	98.75%
	θ_2	-0.019	1.814	98.62%

	q	< 0.0001	0.033	96.40%
	ω	< 0.0001	0.173	91.65%
ARMA(1, 2)	α_1	0.044	0.843	98.34%
	θ_1	0.053	0.929	93.77%
	θ_2	0.040	0.643	91.93%
	q	< 0.0001	0.034	95.28%
	ω	< 0.0001	0.182	91.82%
ARMA(1, 3)	α_1	0.047	1.053	98.64%
	θ_1	0.089	1.121	91.13%
	θ_2	0.034	0.779	90.66%
	θ_3	0.073	0.649	87.15%
	q	-0.001	0.042	96.24%
	ω	-0.001	0.190	92.16%
ARMA(2, 3)	α_1	-0.041	1.951	98.39%
	α_2	0.098	1.461	96.60%
	θ_1	0.118	2.043	97.59%
	θ_2	0.021	0.952	94.36%
	θ_3	0.083	0.776	86.17%
	q	-0.001	0.055	97.33%
	ω	-0.001	0.177	91.80%
ARMA(3, 3)	α_1	0.021	2.042	98.88%
	α_2	0.070	1.720	98.59%
	α_3	-0.006	1.491	99.39%
	θ_1	0.061	2.163	98.47%
	θ_2	0.008	1.798	98.83%
	θ_3	0.095	0.941	89.53%
	q	-0.001	0.036	97.03%
	ω	< 0.0001	0.180	91.90%

Table S2. Model performance measures summary based on a simulation study (simulated sample sizes of *n = 100* observations).

Structure	Parameter	Bias	AIL	Coverage (%)
AR(1)	α	0.006	0.142	94.24%
	q	< 0.0001	0.001	95.34%
	ω	< 0.0001	0.075	93.83%
MA(1)	θ	0.004	0.167	94.92%
	q	< 0.0001	0.001	90.81%
	ω	< 0.0001	0.074	92.73%
ARMA(1, 1)	α	0.008	0.240	95.43%
	θ	0.01	0.299	95.50%
	q	< 0.0001	0.005	94.94%
	ω	< 0.0001	0.082	93.28%
AR(2)	α_1	0.003	0.161	93.59%
	α_2	0.006	0.166	93.69%
	q	< 0.0001	0.001	93.93%
	ω	< 0.0001	0.074	92.59%

AR(3)	α_1	0.004	0.176	94.31%
	α_2	0.006	0.167	94.49%
	α_3	0.005	0.176	94.75%
	q	< 0.0001	0.001	94.77%
	ω	< 0.0001	0.075	92.93%
MA(2)	θ_1	0.001	0.160	95.98%
	θ_2	0.002	0.185	96.56%
	q	< 0.0001	0.001	93.63%
	ω	< 0.0001	0.074	92.58%
MA(3)	θ_1	0.025	0.167	84.19%
	θ_2	0.006	0.191	86.67%
	θ_3	0.031	0.193	84.83%
	q	< 0.0001	0.001	94.04%
	ω	< 0.0001	0.075	92.77%
ARMA(2, 1)	α_1	-0.006	0.942	97.60%
	α_2	0.01	0.643	97.28%
	θ	0.014	0.952	98.32%
	q	< 0.0001	0.004	95.04%
	ω	< 0.0001	0.078	93.76%
ARMA(2, 2)	α_1	0.018	0.740	96.50%
	α_2	-0.013	0.604	95.25%
	θ_1	-0.004	0.743	98.13%
	θ_2	0.027	0.357	91.90%
	q	< 0.0001	0.005	95.95%
	ω	< 0.0001	0.084	93.65%
ARMA(3, 1)	α_1	0.009	0.890	98.04%
	α_2	0.004	0.617	97.88%
	α_3	0.003	0.265	95.12%
	θ	0.001	0.915	98.08%
	q	< 0.0001	0.003	95.56%
	ω	< 0.0001	0.081	94.00%
ARMA(3, 2)	α_1	0.057	1.253	96.84%
	α_2	-0.024	0.945	96.85%
	α_3	-0.015	0.794	97.71%
	θ_1	-0.045	1.255	97.66%
	θ_2	0.022	0.892	94.74%
	q	< 0.0001	0.006	96.76%
	ω	< 0.0001	0.090	93.91%
ARMA(1, 2)	α_1	0.015	0.334	96.65%
	θ_1	0.011	0.377	96.20%
	θ_2	0.017	0.288	93.98%
	q	< 0.0001	0.010	96.23%
	ω	-0.001	0.093	94.31%
ARMA(1, 3)	α_1	0.010	0.397	98.48%
	θ_1	0.049	0.443	86.76%
	θ_2	0.018	0.320	83.76%
	θ_3	0.058	0.285	80.57%

	q	< 0.0001	0.013	95.92%
	ω	< 0.0001	0.101	94.57%
ARMA(2, 3)	α_1	-0.013	1.022	98.30%
	α_2	0.019	0.764	97.84%
	θ_1	0.051	1.028	94.00%
	θ_2	0.011	0.384	87.47%
	θ_3	0.056	0.402	80.15%
	q	< 0.0001	0.008	97.08%
	ω	< 0.0001	0.090	94.01%
ARMA(3, 3)	α_1	0.021	1.082	97.26%
	α_2	0.003	0.888	97.57%
	α_3	-0.013	0.693	98.21%
	θ_1	0.020	1.097	93.41%
	θ_2	0.005	0.755	94.03%
	θ_3	0.068	0.421	79.07%
	q	< 0.0001	0.011	97.49%
	ω	< 0.0001	0.097	94.38%

Table S3. Model performance measures summary based on a simulation study (simulated sample sizes of $n = 500$ observations).

Structure	Parameter	Bias	AIL	Coverage (%)
AR(1)	α	0.003	0.101	94.79%
	q	< 0.0001	0.001	93.28%
	ω	< 0.0001	0.057	92.73%
MA(1)	θ	0.001	0.116	95.61%
	q	< 0.0001	< 0.0001	92.87%
	ω	< 0.0001	0.055	93.96%
ARMA(1, 1)	α	0.003	0.170	95.50%
	θ	0.006	0.213	96.77%
	q	< 0.0001	0.004	95.61%
	ω	< 0.0001	0.065	94.35%
AR(2)	α_1	0.002	0.115	94.34%
	α_2	0.003	0.118	95.13%
	q	< 0.0001	< 0.0001	94.17%
	ω	< 0.0001	0.056	93.69%
AR(3)	α_1	0.001	0.126	95.28%
	α_2	0.004	0.119	94.49%
	α_3	0.002	0.125	95.55%
	q	< 0.0001	< 0.0001	94.99%
	ω	< 0.0001	0.056	93.87%
MA(2)	θ_1	0.001	0.112	95.84%
	θ_2	0.001	0.129	96.98%
	q	< 0.0001	< 0.0001	93.87%
	ω	< 0.0001	0.055	93.69%
MA(3)	θ_1	0.024	0.118	82.43%
	θ_2	0.005	0.133	84.17%

	θ_3	0.031	0.135	82.23%
	q	< 0.0001	< 0.0001	94.48%
	ω	< 0.0001	0.056	93.63%
ARMA(2, 1)	α_1	-0.012	0.708	97.52%
	α_2	0.012	0.482	96.56%
	θ	0.016	0.711	97.04%
	q	< 0.0001	0.001	95.36%
	ω	< 0.0001	0.059	94.08%
ARMA(2, 2)	α_1	0.015	0.547	92.93%
	α_2	-0.016	0.451	92.19%
	θ_1	-0.006	0.540	94.74%
	θ_2	0.025	0.274	90.62%
	q	< 0.0001	0.002	96.53%
	ω	< 0.0001	0.065	94.02%
ARMA(3, 1)	α_1	0.001	0.674	97.51%
	α_2	0.003	0.464	97.55%
	α_3	0.002	0.200	95.87%
	θ	0.003	0.693	97.27%
	q	< 0.0001	0.001	95.63%
	ω	< 0.0001	0.065	95.75%
ARMA(3, 2)	α_1	0.057	0.906	93.46%
	α_2	-0.038	0.678	93.04%
	α_3	-0.012	0.552	95.24%
	θ_1	-0.050	0.900	94.52%
	θ_2	0.036	0.613	90.61%
	q	< 0.0001	0.002	96.63%
	ω	< 0.0001	0.069	94.60%
ARMA(1, 2)	α_1	0.004	0.234	96.36%
	θ_1	0.009	0.256	96.56%
	θ_2	0.011	0.207	94.88%
	q	< 0.0001	0.006	94.74%
	ω	< 0.0001	0.068	94.19%
ARMA(1, 3)	α_1	0.005	0.268	98.31%
	θ_1	0.041	0.298	84.19%
	θ_2	0.013	0.226	82.09%
	θ_3	0.052	0.208	79.56%
	q	< 0.0001	0.010	96.05%
	ω	< 0.0001	0.080	94.61%
ARMA(2, 3)	α_1	-0.004	0.740	97.56%
	α_2	0.007	0.550	97.14%
	θ_1	0.039	0.740	89.83%
	θ_2	0.008	0.276	84.24%
	θ_3	0.048	0.313	78.20%
	q	< 0.0001	0.004	96.73%
	ω	< 0.0001	0.070	94.49%
ARMA(3, 3)	α_1	0.021	0.779	95.03%
	α_2	-0.004	0.631	95.29%

α_3	-0.014	0.458	96.78%
θ_1	0.015	0.787	88.66%
θ_2	0.002	0.496	88.67%
θ_3	0.065	0.311	75.21%
q	< 0.0001	0.004	97.34%
ω	< 0.0001	0.076	94.81%

Table S4. Model performance measures summary for higher order models based on a simulation study (simulated sample sizes of *n = 1000* observations).
